# Clinical and Genetic Features in *EYA1*-Associated Branchio-Oto Syndrome: Cochlear Nerve Deficiency in Five of Thirteen Patients

**DOI:** 10.3390/diagnostics16152480

**Published:** 2026-08-06

**Authors:** Yirong Niu, Yun Lin, Jiali Yu, Huanhuan Zhao, Yuting Zhao, Jie Chen, Ying Sun, Zeqi An, Mengping Wang, Kun Han, Hao Wu, Yun Li, Zhili Wang, Ying Chen

**Affiliations:** 1 Department of Otolaryngology-Head and Neck Surgery, Shanghai Ninth People’s Hospital, Shanghai Jiao Tong University School of Medicine, Shanghai 200011, China; daisy_3146@sjtu.edu.cn (Y.N.); 317031@sh9hospital.org.cn (Y.L.); 216163@sh9hospital.org.cn (J.Y.); zhh277@sjtu.edu.cn (H.Z.); 322053@sh9hospital.org.cn (J.C.); ent@sh9hospital-ent.com (Y.S.); dran2028@sjtu.edu.cn (Z.A.); 217005@sh9hospital.org.cn (M.W.); 316022@sh9hospital.org.cn (K.H.); wuhao@shsmu.edu.cn (H.W.); 116013@sh9hospital.org.cn (Y.L.); 2 Ear Institute, Shanghai Jiao Tong University School of Medicine, Shanghai 200125, China; 3 Shanghai Key Laboratory of Translational Medicine on Ear and Nose Diseases, Shanghai 200125, China; 4 Department of Audiology & Speech-Language Pathology, College of Health Science and Technology, Shanghai Jiao Tong University School of Medicine, Shanghai 200125, China; molly_z@sjtu.edu.cn

**Keywords:** branchio-oto syndrome, hearing loss, cochlear nerve deficiency, *EYA1*, cochlear implantation

## Abstract

**Background/Objectives**: Branchio-oto syndrome (BOS) is an autosomal dominant disorder primarily associated with pathogenic variants in *EYA1*, mainly characterized by branchial anomalies, auricular abnormalities, and hearing loss. However, the co-occurrence of inner ear malformations in BOS remains understudied, especially severe malformations. This study aimed to investigate the clinical and genetic characteristics of patients with *EYA1*-associated BOS, with emphasis on cochlear nerve deficiency (CND). **Methods**: From January 2020 to April 2026, patients diagnosed with *EYA1*-associated BOS at an otology outpatient clinic in a tertiary hospital were included. Clinical manifestations, audiological assessments, imaging and genetic findings were analyzed. **Results**: Thirteen patients (six females and seven males) from eight unrelated families aged 0.3–58.3 years were enrolled. Branchial cleft fistulas and preauricular pits were each observed in 76.9% (10/13) of patients. The mean pure-tone average was 74.3 ± 20.7 dB HL. Eight *EYA1* variants (four truncating, two large deletions, and two splicing) were identified. Among these, five were novel (c.320_329del, c.518del, c.1307dupT, c.1475+1G>A, and exon 12–18 deletion). CND was detected in 38.5% (5/13) of patients and 26.9% (7/26) of ears. Patients with CND carried either truncating variants (*n* = 3) or large deletions (*n* = 2) of *EYA1*. No CND was observed in patients with splicing variants. **Conclusions**: This study identifies five novel *EYA1* pathogenic variants and suggests that CND may be a relatively common radiologic feature in *EYA1*-associated BOS, particularly among patients with truncating variants or large deletions, although larger studies are needed to confirm this association.

## 1. Introduction

Genetic factors are a major cause of congenital hearing loss, which may occur as a non-syndromic or syndromic disorder [1]. Branchio-oto syndrome (BOS) is a rare autosomal dominant syndromic disorder characterized by branchial anomalies, auricular malformations, and hearing loss [2]. Patients with pathologically confirmed renal structural abnormalities and/or renal functional impairment were classified as branchio-oto-renal syndrome (BORS) [3], whereas those without clinically confirmed renal involvement were classified as BOS. Together, BOS and BORS constitute the branchio-oto-renal spectrum disorder (BORSD) with an estimated prevalence of approximately 1 in 40,000 [4].

BOS/BORS demonstrates marked clinical heterogeneity. The diagnosis of BOS/BORS was established according to the BORSD framework [5]. Within the BORSD framework, patients are classified into typical and atypical phenotypes based on the number and combination of major and minor criteria. Typical BORSD was diagnosed by any of the following criteria: (1) ≥three major criteria (hearing loss, preauricular pits, branchial anomalies, renal anomalies, and auricular deformities); (2) two major criteria and ≥two minor criteria (inner ear anomalies, middle ear anomalies, external auditory canal anomalies, preauricular tags, facial asymmetry, and palatal abnormalities); or (3) one major criterion with an affected first-degree relative meeting the diagnostic criteria [6]. Atypical BORSD was diagnosed by either (1) two major criteria or (2) two major criteria plus one minor criterion [7].

Hearing loss is the most common clinical feature of BOS/BORS, ranging from mild to profound degrees and presenting as conductive, sensorineural, or mixed types [8]. Previous imaging studies have reported multiple temporal bone abnormalities, including ossicular malformations, enlarged vestibular aqueduct, and cochlear hypoplasia [9,10,11,12]. However, these studies have primarily focused on structural abnormalities of the cochlea and middle ear, while abnormalities of the cochlear nerve itself have received limited attention.

As a concurrent feature of BOS/BORS, cochlear nerve deficiency (CND) may lead to more severe hearing loss and poorer prognosis [13]. CND was diagnosed by MRI when the cochlear nerve was small or absent [14]. On CT imaging, it is characterized by a bony cochlear nerve canal diameter < 1.4 mm (cochlear aperture stenosis) [15]. Specifically, across four previous reports, a total of 26 BO/BOR patients were described; however, only 18 had explicitly documented radiological assessments (MRI or CT), which formed the basis of our analysis. CND was identified by MRI in 28.6% (2/7) patients [16,17], and by CT in 27.3% (3/11) patients [18,19], respectively. Taken together, a total of 27.8% (5/18) patients in previous reports showed evidence suggestive of CND. Meanwhile, in a study with a larger sample size (including 435 BOR families), the specific details of the cochlear nerve were not assessed due to the absence of MRI [20]. Therefore, comprehensive radiological evaluation is needed in BOS studies, and the prevalence of CND may have been underestimated.

Several genes including *EYA1*, *SIX1*, and *SIX5* are associated with BOS/BORS [4], among which *EYA1* is the most frequently implicated gene, accounting for approximately 40–75% of cases [7]. Previous studies reported that *EYA1* variants accounted for 66.7% of cases in a Japanese cohort [21] and 52.2% in a Korean cohort [22], whereas *SIX1* variants were identified in 17.9% and 34.8% of cases in the Japanese [21] and Korean cohorts [22], respectively. However, *SIX5* variants are rare, and their pathogenic role remains controversial [23]. These findings indicate that both *EYA1* and *SIX1* are important genetic contributors to BO/BOR syndrome in East Asian populations, with *EYA1* remaining the major causative gene. Genetically, BOS is classified into three subtypes: BOS1 (OMIM #602588) is caused by heterozygous *EYA1* variants; BOS2 (OMIM #120502) maps to chromosome 1q31 with its causative gene yet to be identified; and BOS3 (OMIM #608389) results from *SIX1* variants.

In the present study, we analyzed thirteen patients with *EYA1*-associated BOS (BOS1) from eight unrelated Chinese families and systematically reviewed their clinical, audiological, radiological, and genetic findings. Using high-resolution temporal bone CT and internal auditory canal MRI combined with audiological and genetic assessments, we aimed to systematically characterize the radiological spectrum of BOS1 and to investigate the occurrence of CND and its potential association with genetic variants and auditory outcomes.

## 2. Materials and Methods

### 2.1. Study Design and Participants

This study was conducted at the otology outpatient clinic in Shanghai Ninth People’s Hospital, Shanghai Jiao Tong University School of Medicine, a tertiary otology referral center, between January 2020 and April 2026. We performed a systematic retrospective review of all hearing-impaired patients with *EYA1* variants detected through genetic testing during this period. The clinical diagnosis was based on complete and available clinical records, including medical history, physical examination, audiological assessments, and radiological evaluations. The study was approved by the Institutional Review Board of Shanghai Ninth People’s Hospital, Shanghai Jiao Tong University School of Medicine (No. SH9H-2019-T161-2). Written informed consent was obtained from all adult participants and from the legal guardians of pediatric patients before enrollment.

In this study, diagnoses were determined according to the BORSD diagnostic criteria [6]. Phenotypes were classified as typical or atypical based on the constellation of clinical features [7]. Patients were further categorized as BORS or BOS depending on the presence or absence of clinically confirmed renal involvement. Further details are provided in the Supplementary Material.

### 2.2. Clinical Characteristics

A comprehensive clinical evaluation was performed for all enrolled patients. Medical history was reviewed, including age at onset and progression of hearing loss, newborn hearing screening results when available, previous hearing interventions, history of ototoxic drug or noise exposure, and family history of hearing loss. Physical and otoscopic examinations were performed by experienced clinicians.

Clinical features associated with BOS/BORS were assessed, including branchial cleft fistulas, preauricular pits, external ear anomalies, and facial paralysis. Renal involvement was evaluated based on renal ultrasonography and available medical records.

### 2.3. Audiological Assessments

Audiological evaluations were performed for all patients, including otoscopic examination, tympanometry and wideband tympanometry, pure-tone audiometry, and auditory brainstem response (ABR) testing when appropriate. For each patient, wideband tympanometry (WBT), including measurements at 226 and 1000 Hz, was routinely performed before hearing threshold assessment. Hearing thresholds were evaluated using click-evoked auditory brainstem response (click-ABR; Interacoustics, Middelfart, Denmark) and behavioral audiometry (Otometrics, Taastrup, Denmark) at 0.5, 1, 2, and 4 kHz. For ears that underwent hearing aids (HA) and cochlear implantation (CI), aided PTA was calculated using the same frequency average when available. They were graded as mild (20.0 to <35.0 dB HL), moderate (35.0 to <50.0 dB HL), moderately severe (50.0 to <65.0 dB HL), severe (65.0 to <80.0 dB HL), profound (80.0 to <95.0 dB HL) and complete (95.0 dB HL or greater) hearing loss, following the 2021 WHO Guidelines.

Speech recognition scores (SRSs) were assessed in quiet using Mandarin Chinese monosyllabic and disyllabic word recognition lists. Speech stimuli were presented in the sound field at a presentation level of 60.0 dB HL, and each test list consisted of 20.0 words. Participants were instructed to repeat each word verbally, and responses were scored by the examiner. The final SRS was expressed as the percentage of correctly identified words. All assessments were performed using a Madsen Astera audiometer (Natus Sensory, Gort, Ireland).

### 2.4. Radiological Evaluations

High-resolution computed tomography (HRCT) scans of temporal bone were performed using a 128-slice spiral CT scanner (Siemens, SOMATOM Definition Flash, Forchheim, Germany). HRCT was performed to assess external, middle, and inner ear structures, including the external auditory canal, ossicular chain, cochlea, vestibule, semicircular canals, vestibular aqueduct, and internal auditory canal. Magnetic resonance imaging (MRI) examinations were performed using a 3.0-T MAGNETOM Prisma MRI system (Siemens Healthineers, Erlangen, Germany) equipped with a 64-channel head and neck coil. High-resolution T2-weighted images of the internal auditory canals were acquired using a three-dimensional SPACE sequence with a slice thickness of 0.5 mm. Oblique sagittal scans were also performed for both sides. All imaging results were independently assessed by at least three experienced radiologists or otolaryngologists to reach a final diagnosis.

### 2.5. Genetic Analysis

Genomic DNA was extracted from peripheral blood samples using a Genomic DNA Extraction Kit (MyGenostics Inc., Beijing, China). Exome sequencing was performed to identify candidate pathogenic variants. Briefly, sequencing libraries were prepared using a standard library construction kit (MyGenostics Inc.), including DNA fragmentation, end repair, adenylation, and adapter ligation. Exonic regions and exon–intron boundaries, extending 50 bp into adjacent intronic regions, were captured using the GenCap capture kit (MyGenostics Inc.). High-throughput sequencing was performed on an Illumina NextSeq 550 platform (Illumina, San Diego, CA, USA).

Raw sequencing data were assessed using FastQC v0.11.9, and adapter sequences were trimmed with Cutadapt v1.16. Clean reads were aligned to the human reference genome GRCh37/hg19 using BWA v0.7.10. The resulting alignment files were processed using Samtools v1.2 and Picard v2.22.0 for format conversion and duplicate removal. Variant calling was performed using the Genome Analysis Toolkit (GATK v4.0.8.1), and variant annotation was conducted using ANNOVAR (version: 2018-04-16). Variants were described according to the Human Genome Variation Society (HGVS) nomenclature.

Candidate *EYA1* variants identified by exome sequencing were validated by Sanger sequencing. Primers were designed using Primer3 to amplify the regions containing the variants of interest. PCR products were sequenced using the BigDye Terminator v3.1 Cycle Sequencing Kit (Thermo Fisher Scientific, Waltham, MA, USA) on an ABI 3130XL Genetic Analyzer (Applied Biosystems, Waltham, MA, USA). The *EYA1* reference transcript NM_000503.6 was used for variant annotation. When DNA samples from family members were available, segregation analysis was performed by Sanger sequencing.

Quantitative PCR (qPCR) was performed to validate copy number variants involving *EYA1* and to assess segregation in available family members. qPCR reactions were conducted in triplicate using TB Green Premix Ex Taq (Tli RNaseH Plus; Takara Bio Inc., Kusatsu, Shiga, Japan) on a LightCycler 480 System (Roche Diagnostics, Mannheim, Germany). The cycling conditions were 95 °C for 30 s, followed by 40 cycles of 95 °C for 5 s and 55 °C for 30 s. Specific primers were designed using NCBI Primer-BLAST (https://www.ncbi.nlm.nih.gov/tools/primer-blast/, accessed on 2 January 2026). Relative copy number was calculated using the 2^−ΔΔCt^ method and normalized to an internal reference gene.

Detected *EYA1* variants were classified according to the American College of Medical Genetics and Genomics and the Association for Molecular Pathology (ACMG/AMP) guidelines. All *EYA1* variants included in this study were classified as pathogenic or likely pathogenic based on the original genetic testing reports and subsequent review.

### 2.6. Electrophysiological Assessments

Auditory cortical responses were assessed using multichannel electroencephalography (EEG; Brain Products, Gilching, Germany) during a mismatch negativity (MMN) paradigm. Recordings were performed in a quiet environment. Pure-tone stimuli were presented in the sound field at 70 dB. Standard tones of 500 Hz and deviant tones of 1000 Hz were presented in a pseudorandom sequence with probabilities of 80% and 20%, respectively. Each stimulus had a duration of 500 ms, with an inter-stimulus interval of 300 ms. Stimuli were delivered through two loudspeakers positioned approximately 100 cm from the participant using E-Prime 2.0.

EEG data were recorded using electrodes positioned according to the International 10–20 system. The recording window ranged from 200 ms before stimulus onset to 600 ms after stimulus onset. EEG signals were band-pass filtered between 1 and 30 Hz. Epochs with amplitudes exceeding ±20 μV were rejected offline, and the remaining responses were averaged to generate the individual grand-averaged waveform.

### 2.7. Statistical Analysis

Continuous variables were compared using the Mann–Whitney U test. Categorical variables were compared using Fisher’s exact test. All statistical tests were two-sided, and *p* values < 0.05 were considered statistically significant.

## 3. Results

### 3.1. Clinical Features and Hearing Phenotypes

**Figure 1:**
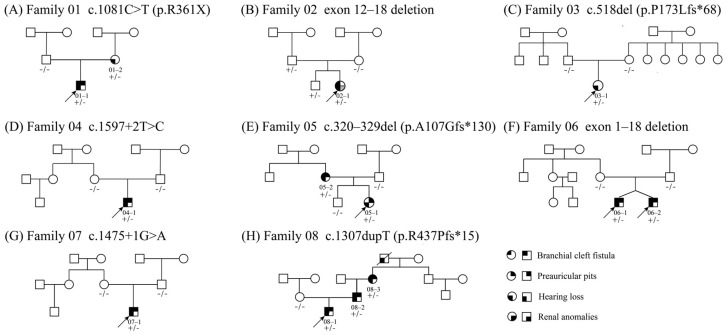
Pedigrees and *EYA1* variants of the eight unrelated families with *EYA1*-associated branchio-oto syndrome (**A**–**H**). Arrows indicate the proband. Shading indicates suspicious renal involvement in patient F02-1 (mild renal pelvic separation [5 mm] with normal renal function). Genotypes are shown below the symbols for individuals. Wild type: −/−; heterozygous: +/−. Individuals without genotype labels were not genetically tested.

From January 2020 to April 2026, a total of thirteen BOS patients from eight unrelated Chinese families with genetically confirmed *EYA1* variants at our center were included. Seven of them were males and six were females (Figure 1). Ages at the initial follow-up ranged from 0.3 to 58.3 years, with a mean of 14.92 ± 18.70 years. Branchial cleft fistulas were identified in 76.9% of patients (10/13), and preauricular pits were also present in 76.9% of patients (10/13). External ear anomalies were observed in 38.5% of patients (5/13).

During our follow-up visit, no definite renal function impairment was proved in any patient. Four cochlear implant patients had normal renal function test results at our hospital, including patient F02-1, who showed a mild renal pelvic separation (5 mm) on a single ultrasound examination; given concurrent normal renal function test results, no further imaging follow-up was scheduled in the short term. The other nine patients showed basically normal renal function test results and/or renal ultrasound findings. All patients in our study were phenotypically classified as BOS and further designated as BOS1 based on genetic testing results.

All patients presented with hearing loss. Initial and latest follow-up hearing assessments were summarized in Table 1. Behavioral audiometry was available in 92.3% of patients (12/13), whereas patient F07-1 at 1.9y was evaluated using auditory brainstem response (ABR) only and was excluded from PTA-based statistical analysis. In patients with available behavioral thresholds, the better-ear PTA ranged from 41 to 111 dB HL, with a mean of 74.3 ± 20.7 dB HL (*n* = 12). According to the World Health Organization 2021 classification, hearing loss based on the better-hearing ear was moderate in 8.3% of patients (1/12), moderately severe in 16.7% (2/12), severe in 58.3% (7/12), and complete in 16.7% (2/12). Tympanometry was performed in all patients, showing type A tympanograms in 76.9% of patients (10/13) and type B tympanograms in 23.1% of patients (3/13).

In addition to the common manifestations of BOS1, several patients showed less commonly reported findings. Atrial septal defect was identified in 23.1% of patients (3/13) during cardiac evaluation. In addition, the twin brothers, patients F06-1 and F06-2, both presented with peripheral facial nerve palsy. Imaging examinations showed facial nerve structural abnormalities in both patients, and surgical intervention was therefore not performed.

### 3.2. Radiological Findings

All patients underwent temporal bone HRCT and internal auditory canal MRI to evaluate middle and inner ear structures, with particular focus on the cochlear nerve. Radiological abnormalities were identified across multiple anatomical regions, involving the external auditory canal, middle ear, ossicular chain, cochlea, vestibular aqueduct, semicircular canals, internal auditory canal, and bony cochlear nerve canal.

HRCT revealed various inner ear malformations, including cochlear hypoplasia, enlarged vestibular aqueduct, and semicircular canal dysplasia. Ossicular chain abnormalities were observed in 38.5% of patients (5/13). Enlarged vestibular aqueduct was identified in 30.8% of patients (4/13). In patient F02-1, abnormal cochlear morphology involving fusion of the apical and middle turns was observed. MRI of the internal auditory canal was used to assess the cochlear nerve. CND was identified in 38.5% of patients (5/13), involving 26.9% of ears (7/26). Bilateral CND was present in 15.4% of patients (2/13), and unilateral CND was observed in 23.1% of patients (3/13). All unilateral cases involved the right ear in this study. Ear-level analysis showed mean PTA thresholds of 95.1 ± 21.9 dB HL in ears (*n* = 7) with CND and 80.3 ± 23.1 dB HL in ears without CND (*n* = 17); the difference was not significant (*p* = 0.162). Patients with bilateral CND exhibited severe-to-profound hearing loss and underwent CI for auditory rehabilitation. Among patients with unilateral CND, asymmetric hearing loss was observed in patient F08-1, with poorer hearing thresholds on the affected side.

**Figure 2:**
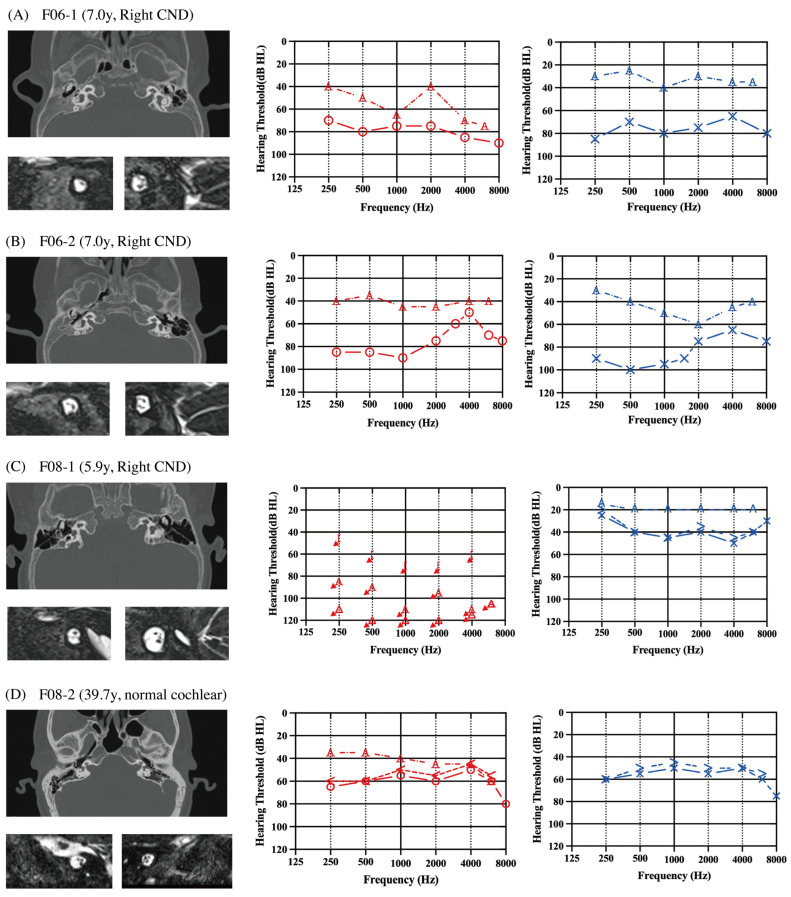
Audiological and imaging findings in Family 06 and Family 08. Axial temporal bone HRCT images, internal auditory canal MRI, pure-tone ear audiograms are shown for patients F06-1 (**A**), F06-2 (**B**), F08-1 (**C**), and F08-2 (**D**). All audiometric symbols conform to American Speech-Language-Hearing Association standards: red circle (○) denotes right-ear air conduction, blue cross (×) denotes left-ear air conduction, red leftward triangle (<) denotes right-ear bone conduction, blue rightward triangle (>) denotes left-ear bone conduction, downward-pointing arrow (↓) denotes no response, and symbol “A” denotes aided PTA. The symbol A denotes aided PTA. Bone conduction thresholds were not shown for patients F06-1 and F06-2, as they were unable to complete contralateral (right-sided) masking during pure-tone bone conduction audiometry.

In this study, intrafamilial phenotypic variability was observed among individuals carrying identical *EYA1* variants (Figure 2). In the twin family (Family 06), both siblings harbored the same pathogenic variant and exhibited concordant phenotypes, including similar degrees of hearing loss and the presence of CND. In contrast, phenotypic discordance was observed in Family 08 (Figure 2). The proband (F08-1) presented with unilateral CND accompanied by severe to profound hearing loss, whereas his father (F08-2), despite carrying the same *EYA1* variant, showed no evidence of CND and demonstrated moderately severe hearing loss.

### 3.3. Genetic Findings

All patients carried pathogenic or likely pathogenic variants in *EYA1*. Heterozygous *EYA1* variants were identified in all thirteen patients from the eight unrelated families (Table 1). A total of eight distinct *EYA1* variants were detected, including one nonsense variant, two large deletion variants, three frameshift variants, and two splicing variants. Among the eight distinct *EYA1* variants, five were novel and had not been previously reported in the literature or public databases, including ClinVar and HGMD. These novel variants included c.320_329del, c.518del, c.1307dupT, c.1475+1G>A, and exon 12-18 deletion. Of note, the c.1475+1G>T and c.1475+1G>C variants of splicing site have already been reported [24,25], but the c.1475+1G>A variant remains novel.

Parental analysis revealed four de novo variants among the eight families (50.0%, 4/8), including the frameshift variant c.518del (p.P173Lfs*68) in Family 03, the splicing variant c.1597+2T>C in Family 04, the exon 1–18 deletion in Family 06, and the splicing variant c.1475+1G>A in Family 07. In contrast, the p.R361X variant identified in Family 01 was confirmed to be maternally inherited, as the patient’s mother carried the same heterozygous *EYA1* variant. Large genomic deletions were identified in two families. In Family 06, the twin brothers carried a heterozygous deletion spanning the entire coding region of *EYA1* (exon 1–18). In Family 02, the proband (F02-1), who presented with branchial cleft fistula, hearing loss, and right renal pelvis separation, carried a heterozygous deletion involving exon 12–18 of *EYA1*. During genetic validation, the proband’s father and brother were incidentally found to carry the same exon 12–18 deletion. However, none of the major features of BORSD: hearing loss, renal impairment, preauricular pits, branchial fistulas, or auricular deformities have been found. Therefore, they could not be diagnosed with BOS or BOS1, and were not included in our analysis.

Further integration of genetic and radiological findings showed that all five patients with CND harbored either truncating variants or large deletions of *EYA1*, including p.R361X, p.P173Lfs*68, p.R437Pfs*15, and exon 1–18 deletion. However, not all individuals carrying truncating variants or large deletions exhibited CND, suggesting incomplete penetrance of this radiological phenotype. Overall, CND was observed in 45.5% (5/11) of patients with truncating variants or large deletions, whereas no CND was identified in patients carrying splicing variants. Exploratory Fisher’s exact test showed no significant association between variant category and CND occurrence (*p* = 0.487), likely due to the limited sample size. These findings suggest that highly disruptive *EYA1* variants may contribute to cochlear nerve developmental abnormalities, although larger studies are required to validate this potential association.

**Table 1:** Demographic, clinical, auditory manifestations in BOS patients with *EYA1* variants.

Families	Patients	Sex	Age (y)	Initial Hearing (PTA) ^a^		Last Hearing (PTA) ^a^				Hearing Intervention		Cochlear Nerve Deficiency	Major Diagnostic Criteria Other than HL ^b^				*EYA1* Variant ^d^	De Novo ^e^	Literature
				L	R	L	R	Aided L	Aided R	L	R		BA	PP	RA	AD			
1	01-1	M	1.7	/	/	118	113	/	70	/	CI	Bilateral	+	+	−	+	c.1081C>T (p.R361X)	−	Spruijt et al., 2006 [26]
	01-2	F	27.4	/	/	86	74	/	/	/	/	None	−	−	−	−			
2	02-1	F	2.7	/	/	108	79	NA	39	CI	HA	None	+	−	susp. ^c^	−	exon 12-18 deletion	−	This study
3	03-1	F	30.3	/	/	65	96	38	42	CI	HA	Bilateral	−	−	−	+	c.518del (p.P173Lfs*68)	+	This study
4	04-1	M	1.6	/	/	60	66	/	41	/	HA	None	+	+	−	−	c.1597+2T>C	+	Ben-Moshe et al., 2023 [27]
5	05-1	F	4.7	120	120	120	111	40	58	CI	HA	None	−	+	−	−	c.320_329del (p.A107Gfs*130)	−	This study
	05-2	F	28.6	114	74	118	74	/	38	/	HA	None	+	+	−	−			
6	06-1	M	1.2	60 *	97 *	73	81	23	23	HA	HA	Right	+	+	−	+	exon 1-18 deletion	+	Li et al., 2021 [28]
	06-2	M	1.2	65 *	60 *	84	75	23	23	HA	HA	Right	+	+	−	+			
7	07-1	M	1.9	/	/	55 *	50 *	/	/	/	/	None	+	+	−	−	c.1475+1G>A	+	This study
8	08-1	M	0.3	30 *	80 *	41	118	29	95	HA	HA	Right	+	+	−	+	c.1307dupT (p.R437Pfs*15)	−	This study
	08-2	M	34.1	53	53	53	56	/	39	/	HA	None	+	+	−	−			
	08-3	F	58.3	/	/	73	89	/	/	/	/	None	+	+	−	−			

^a^ Asterisks (*) denote click-ABR results. ^b^ A typical BORSD diagnosis was established when patients fulfilled any of the following criteria: (1) ≥3 major criteria; (2) two major criteria and ≥2 minor criteria (including inner ear anomalies, middle ear anomalies, external auditory canal anomalies, preauricular tags, facial asymmetry, and palatal abnormalities); or (3) one major criterion with an affected first-degree relative meeting the diagnostic criteria. An atypical BORSD diagnosis was established when patients fulfilled either of the following criteria: (1) two major criteria; or (2) two major criteria plus one minor criterion. ^c^ Suspicious renal involvement: patient F02-1 showed mild renal pelvic separation (5 mm); however, renal function tests remained normal. Therefore, the patient was classified as BOS rather than BORS. ^d^ All variants were heterozygous. ^e^ “+” in the de novo column indicates a confirmed de novo variant; “−” indicates the variant was inherited from a parent; “NA” indicates parental verification was not performed or parental samples were unavailable. Abbreviations: HA: hearing aids; CI: cochlear implantation; HL: Hearing Loss; BA: Branchial Anomalies; PP: Preauricular Pits; RA: Renal Anomaly; AD: Auricular Deformity.

### 3.4. Hearing Intervention and Auditory Rehabilitation Outcomes

Patients were stratified into CND and non-CND groups based on internal auditory canal MRI findings. Hearing outcomes after intervention were analyzed at the ear level and compared by CND (7 ears) and non-CND (17 ears) groups. The mean unaided PTA was 95.1 ± 21.9 dB HL in CND and 80.3 ± 23.1 dB HL in non-CND ears. Six CND ears and ten non-CND ears received hearing intervention. Among CND ears, four ears received HAs, with aided PTA ranging from 23.0 to 95.0 dB HL (45.8 ± 34.0 dB HL); two ears underwent CIs, achieving postoperative aided PTA of 70.0 and 38.0 dB HL, respectively. Among non-CND ears, eight ears received HAs, with aided PTA ranging from 23.0 to 58 dB HL (36.3 ± 11.4 dB HL); two ears underwent CIs, with postoperative aided PTA data available for one ear, achieving 40.0 dB HL.

**Figure 3:**
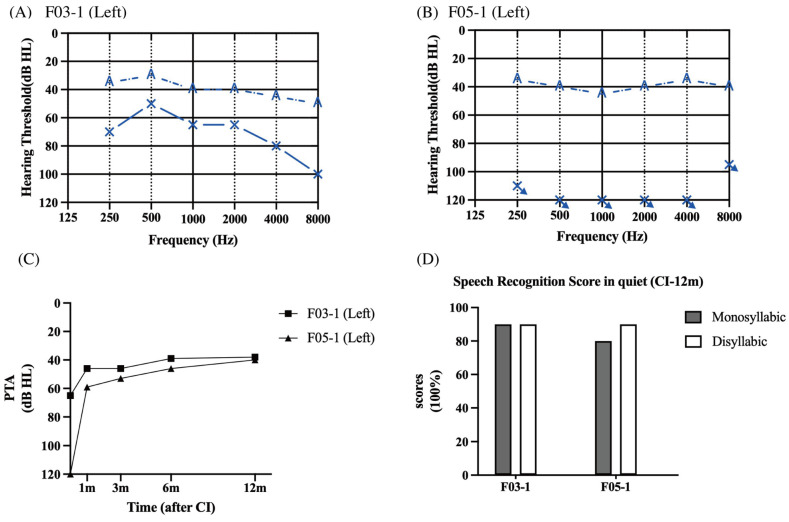
Hearing outcomes in patients with and without CND. Unaided and CI-aided audiograms of patient F03-1 (**A**) with bilateral CND and patient F05-1 (**B**) without CND 12 months after CI. All audiometric symbols conform to American Speech-Language-Hearing Association standards: blue crosse (×) denotes left-ear air conduction, downward-pointing arrow (↓) denotes no response, and symbol “A” represents the aided PTA. (**C**) Changes in aided PTA before and after CI. (**D**) Speech recognition scores in quiet at twelve months after CI, including monosyllabic and disyllabic word recognition scores. All patients illustrated in this figure used CI for auditory rehabilitation.

Patient F03-1 was diagnosed with bilateral CND. Preoperative pure-tone audiometry revealed a PTA of 65.0 dB HL in the left ear and 96.0 dB HL in the right ear (Figure 3A). After HA fitting, monosyllabic word recognition scores in a sound field at a presentation level of 60.0 dB HL were 25% in the left ear and 80% in the right ear. Based on the speech recognition assessments, CI on the left side was decided by experienced physicians. At the 12-month follow-up, the aided PTA of the implanted ear (left) improved to 40.0 dB HL (Figure 3C). Speech recognition testing showed that both monosyllabic and disyllabic word scores reached 90% (Figure 3D). Patient F05-1 presented with bilateral profound hearing loss (Figure 3B). After HA fitting, monosyllabic word recognition scores in a sound field at a presentation level of 60 dB HL were 0% bilaterally. Left-sided CI was subsequently performed. At the 12-month follow-up, the aided PTA of the implanted ear (left) was 40.0 dB HL (Figure 3C). Speech recognition testing showed scores of 80% for monosyllabic words and 90% for disyllabic words (Figure 3D).

**Figure 4:**
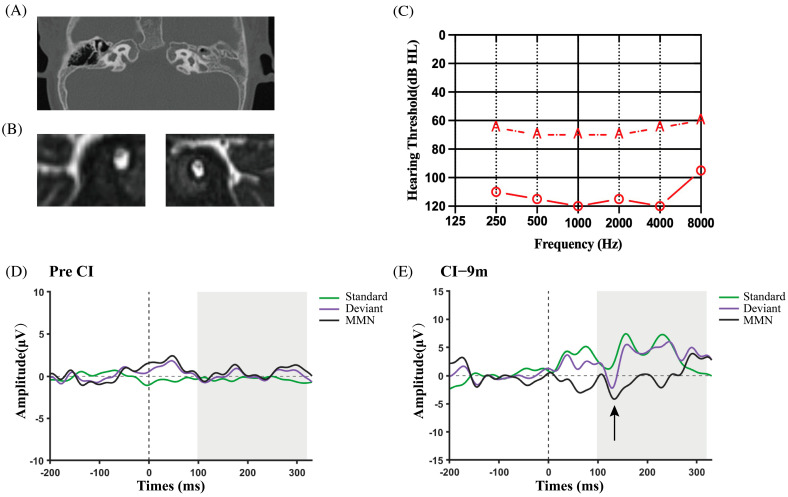
Assessments of patient F01-1 with bilateral CND. (**A**) Axial temporal bone CT image. (**B**) Internal auditory canal MRI. (**C**) Unaided and aided audiogram nine months after right CI. All audiometric symbols conform to American Speech-Language-Hearing Association standards: red circle (○) denotes right-ear air conduction, and symbol “A” represents the aided PTA. (**D**) Auditory response and mismatch negativity (MMN) recording before CI and (**E**) nine months after right CI. The gray region shows a significantly different time (from 98 to 320 ms) between the standard and deviant conditions. The prominent negative deflection (indicated by the black arrow in E) represents the MMN response.

Patient F01-1 had bilateral CND (Figure 4A,B). Before CI, no clear cortical auditory evoked potentials or MMN responses were recorded under unaided acoustic stimulation at 70.0 dB HL (Figure 4D). Nine months after right-sided CI activation, cortical auditory evoked potentials and MMN responses were recorded under a 70.0 dB HL tone-burst paradigm (Figure 4E). The FCz response showed an amplitude of 3.97 μV and a latency of 101 ms, while the averaged response showed an amplitude of 13.62 μV and a latency of 131 ms.

## 4. Discussion

### 4.1. CND in EYA1-Associated BOS

The prevalence of CND has only been reported in a few previous studies with small sample sizes. One study demonstrated that the mean cochlear aperture diameter was smaller in patients with BORS (1.42 ± 0.37 mm, *n* = 38) than in normal controls (2.05 ± 0.24 mm, *n* = 21), suggesting that cochlear aperture stenosis may be relatively common in BORS; however, the specific prevalence of CND remained unclear [29]. In the present study, CND was identified in 38.5% of patients (5/13) and 26.9% of ears (7/26). In the patient-level analysis, this prevalence showed no significant difference compared to prior reports (27.8%, 5/18; *p* = 0.70) [16,17,18,19]. Similarly, in the ear-level analysis, our finding showed no significant difference compared to prior reports (33.3%, 4/12; *p* = 0.76) [16,17]. Given the limited sample size and the potential referral bias inherent to our tertiary referral center, further studies with larger cohorts are warranted to clarify the true prevalence of CND in the BORSD population. Moreover, as CT and MRI become more widely adopted in clinical practice, future investigations may help to further elucidate this issue.

### 4.2. Phenotypic Heterogeneity in EYA1-Associated BOS

Genetic evidence from previous BOS/BORS studies has shown that pathogenic *EYA1* variants are highly heterogeneous, with truncating variants accounting for a major proportion of reported cases [5,30]. Data from the HGMD database indicate that most pathogenic or likely pathogenic *EYA1* variants associated with BOS are truncating variants, whereas missense variants, splicing variants, and large deletions account for only 14.4%, 16.9%, and 9.9%, respectively [31]. In the present study, truncating variants were identified in 61.5% of patients (8/13), splicing variants in 15.4% (2/13), and large deletions in 23.1% (3/13), whereas no missense variants were detected. In this cohort, all five patients with CND carried a truncating variant or large deletion; however, the sample was insufficient to establish a genotype–phenotype association. Among the five previously reported BO/BOR patients with CND, three did not undergo genetic testing [16,17,18,19], while two carried the *EYA1* splicing variant c.967-2A>G [19]. The de novo variant ratio was 50.0% (4/8) in our study. Two of the four de novo variants had not been previously reported (c.518del and c.1475+1G>A). Among the five patients from these four families, two patients in Family 06 and one patient in Family 03 presented with CND. These findings suggest that the development of CND is not exclusively dictated by the type of *EYA1* variant and no clear association has been established between *EYA1* variant type and the occurrence of CND.

*EYA1* mutations can exhibit incomplete penetrance and highly variable expressivity, even within the same pedigree [32]. In our study, the proband (F08-1) presented with unilateral CND and severe-to-profound hearing loss, whereas his father (F08-2), who also had BOS and carried the same variant, exhibited a milder auditory phenotype without evidence of CND. Moreover, in Family 02, the *EYA1* exon 12–18 deletion was identified in both the proband (F02-1) and two other relatives who had not been confirmed as having BOS. Previous studies have established that *EYA1* mutations exhibit incomplete penetrance and highly variable expressivity [5,33,34]. This may be due to the haploinsufficiency of this gene: different tissues require distinct *Eya1* dosage thresholds during development to maintain normal morphogenesis [35]. Furthermore, heterozygous *Eya1* mouse models similarly exhibit low penetrance, milder phenotypes compared to humans, further validating this mechanism [36]. Larger cohorts and functional investigations are needed to better define the potential contributing factors, including genetic modifiers, environmental influences, and developmental variability.

### 4.3. Renal Evaluation in EYA1-Associated BOS Patients

BO and BOR represent variable phenotypic manifestations within the BORSD spectrum, and definite renal involvement constitutes the key clinical feature distinguishing BOR from BOS [6]. However, the interpretation of renal manifestations in BORSD can be challenging because of incomplete penetrance and variable expressivity, particularly in individuals presenting with subtle or isolated renal imaging abnormalities [32]. Moreover, the age of patients and the follow-up duration are also critical considerations, as younger patients may not yet have developed renal function impairment at the time of assessment.

In our study, the majority of patients (8/13) were younger than 5 years at presentation. One patient in our study exhibited mild right renal pelvis separation on ultrasonography, but her renal function remained normal. This incidental finding precluded the need for short-term repeat imaging; however, continued long-term surveillance is warranted. Therefore, all patients in this study were classified as BOS at the current assessment. Nevertheless, it is important to emphasize that for individuals meeting the diagnostic criteria for BORSD, especially those with confirmed *EYA1* defects, continued renal surveillance is essential, due to the age-dependent penetrance of *EYA1*-associated nephropathy [6].

Even in patients without structural renal abnormalities, *EYA1*-associated renal damage may emerge and progress over time. A previous study of a BOR family with the *EYA1* splicing variant c.1050+5G>A demonstrated that the 4-year-old proband presented with glucocorticoid-resistant proteinuria. Over an 8-year follow-up period, the proband’s mother and brother, who initially exhibited no structural renal abnormalities, gradually developed proteinuria with progressive glomerular filtration rate decline [31]. In another study, a 27-year follow-up of a three-generation BOR family documented that four of ten genetically affected members developed end-stage renal disease at ages 36, 14, 17, and 17 years [32].

### 4.4. Hearing Outcomes in Patients with CND (HA and CI)

Overall, the hearing intervention outcomes in the present study showed that both HA and CI can provide auditory benefit in patients with *EYA1*-associated BOS, which is consistent with previous reports [30]. Approximately 80% of patients gained auditory benefits from HA in the cohorts, yet neither study reported aided PTA [4,30]. CI was less frequently described, covering five patients who achieved post-implantation PTAs between 40 and 70 dB HL [37]. However, in our case series, patients with *EYA1*-associated BOS and concomitant CND generally presented with severe hearing loss and showed variable outcomes after auditory rehabilitation. Among patients with CND, HA-aided PTA ranged from 23.0 to 101.0 dB HL, and CI-aided PTA ranged from 38.0 to 70.0 dB HL. Patients with relatively milder baseline hearing loss appeared to achieve better auditory outcomes, whereas those with more severe hearing loss showed more limited benefit. However, given the small sample size, variable ages at intervention, and different HA/CI intervention strategies, these observations should be considered exploratory and require validation in larger cohorts. Comprehensive pre- and post-intervention assessments remain important for individualized auditory rehabilitation in patients with BOS1 and CND.

## 5. Strengths and Limitations

This study systematically evaluated CND in patients with *EYA1*-associated branchio-oto syndrome, providing clinically relevant evidence on the radiological spectrum of this under-recognized phenotype. To our knowledge, this study represents one of the few reported BOS/BORS case series with dedicated assessment of cochlear nerve status.

However, several limitations should be acknowledged. First, this study was conducted at a tertiary otology referral center, which may introduce selection bias on the prevalence of CND among BOS patients. Patients referred to our center tend to present more severe hearing loss, and the observed prevalence of CND may be overestimated. Multicenter data from primary care settings are required to further investigate BOS in a broader population. Second, the overall sample size is limited, particularly after stratification by genotype and CND status, which restricts robust geno-phenotype analyses. Third, the wide age range of enrolled patients may have influenced the interpretation of hearing outcomes. Therefore, age-related changes in hearing function will be further evaluated through longitudinal studies. Finally, no pathogenic variants in *SIX1* or *SIX5* were identified in this case series; therefore, their potential contribution to CND-associated phenotypes could not be evaluated, and their role in CND pathogenesis remains unknown.

## 6. Conclusions

This study identifies five novel *EYA1* pathogenic variants and suggests that CND may be a relatively common radiologic feature in *EYA1*-associated BOS, particularly among patients with truncating variants or large deletions, although larger studies are needed to confirm this association.

## Data Availability

The data presented in this study are available on request from the corresponding authors. The data are not publicly available due to privacy or ethical restrictions.

## Supplementary Materials

The following supporting information can be downloaded at https://www.mdpi.com/article/10.3390/diagnostics16152480/s1, Table S1: Distribution of Clinical Features in the Study Cohort.

## Abbreviations

The following abbreviations are used in this manuscript:

BOS	Branchio-oto syndrome
BORS	Branchio-oto-renal syndrome
CND	Cochlear nerve deficiency
ABR	Auditory brainstem response
PTA	Pure-tone average
SRS	Speech recognition scores
HA	Hearing aids
CI	Cochlear implantation
HL	Hearing Loss
BA	Branchial Anomalies
PP	Preauricular Pits
RA	Renal Anomaly
AD	Auricular Deformity
HRCT	High-resolution Computed Tomography
MRI	Magnetic Resonance Imaging
GATK	Genome Analysis Toolkit
HGVS	Human Genome Variation Society
qPCR	Quantitative PCR
ACMG	American College of Medical Genetics
AMP	Genomics and the Association for Molecular Pathology
EEG	Electroencephalography
MMN	Mismatch negativity
