# Supplementary material for: Clinical and Genetic Features in EYA1-Associated Branchio-Oto Syndrome: Cochlear Nerve Deficiency in Five of Thirteen Patients
Source: Diagnostics (Basel). 2026 Aug 6;16(15):2480. doi: 10.3390/diagnostics16152480 (PMC13464796; doi:10.3390/diagnostics16152480)
Supplement: Supplementary file 1 [file diagnostics-16-02480-s001.zip › diagnostics-4434081-supplementary.pdf]

Supplemental Table S1. Distribution of Clinical Features in the Study Cohort

Clinical Features	Patients												
	**F01-1**	**F01-2**	**F02-1**	**F03-1***	**F04-1**	**F05-1**	**F05-2**	**F06-1**	**F06-2**	**F07-1**	**F08-1**	**F08-2**	**F08-3**
**Major Critieria**													
Branchial anomalies	+	—	+	—	+	—	+	+	+	+	+	+	+
Hearing loss	+	+	+	+	+	+	+	+	+	+	+	+	+
Preauricular pits	+	+	—	—	+	+	+	+	+	+	+	+	+
Renal anomaly	—	—	susp[a]	—	—	—	—	—	—	—	—	—	—
Auricular deformity	+	—	—	+	—	—	—	+	+	—	+	—	—
**Minor Criteria**													
External auditory canal anomalies	—	—	—	—	—	—	—	—	—	—	—	—	—
Middle ear anomalies	+	—	+	—	—	—	—	+	+	+	—	—	—
Inner ear anomalies	+	—	+	+	+	+	—	+	+	+	+	—	—
Other: facial asymmetry	—	—	—	—	—	—	—	+	+	—	—	—	—

a Suspicious renal involvement: F02-1 showed mild renal pelvic separation (5 mm); however, renal function tests remained normal.

* Patient F03-1 was classified as atypical BOS
